# Genotyping the High Altitude Mestizo Ecuadorian Population Affected with Prostate Cancer

**DOI:** 10.1155/2017/3507671

**Published:** 2017-06-08

**Authors:** Andrés López-Cortés, Alejandro Cabrera-Andrade, Carolina Salazar-Ruales, Ana Karina Zambrano, Santiago Guerrero, Patricia Guevara, Paola E. Leone, César Paz-y-Miño

**Affiliations:** ^1^Centro de Investigación Genética y Genómica, Facultad de Ciencias de la Salud Eugenio Espejo, Universidad Tecnológica Equinoccial, Avenue Mariscal Sucre, 170129 Quito, Ecuador; ^2^Gene Regulation, Stem Cells and Cancer Programme, Centre for Genomic Regulation (CRG), The Barcelona Institute for Science and Technology, Universitat Pompeu Fabra (UPF), Dr. Aiguader 88 Street, 08003 Barcelona, Spain

## Abstract

Prostate cancer (PC) is the second most commonly diagnosed type of cancer in males with 1,114,072 new cases in 2015. The MTHFR enzyme acts in the folate metabolism, which is essential in methylation and synthesis of nucleic acids. MTHFR C677T alters homocysteine levels and folate assimilation associated with DNA damage. Androgens play essential roles in prostate growth. The SRD5A2 enzyme metabolizes testosterone and the V89L polymorphism reduces in vivo SRD5A2 activity. The androgen receptor gene codes for a three-domain protein that contains two polymorphic trinucleotide repeats (CAG, GGC). Therefore, it is essential to know how PC risk is associated with clinical features and polymorphisms in high altitude Ecuadorian mestizo populations. We analyzed 480 healthy and 326 affected men from our three retrospective case-control studies. We found significant association between MTHFR C/T (odds ratio [OR] = 2.2; *P* = 0.009), MTHFR C/T+T/T (OR = 2.22; *P* = 0.009), and PC. The SRD5A2 A49T substitution was associated with higher pTNM stage (OR = 2.88; *P* = 0.039) and elevated Gleason grade (OR = 3.15; *P* = 0.004). Additionally, patients with ≤21 CAG repeats have an increased risk of developing PC (OR = 2.99; *P* < 0.001). In conclusion, genotype polymorphism studies are important to characterize genetic variations in high altitude mestizo populations.

## 1. Epidemiology of Prostate Cancer

Prostate cancer (PC) represents a significant health problem that involves the progressive accumulation of environmental, hormonal, genetic, and hereditary factors [1]. PC is the second most commonly diagnosed type of cancer in males, representing ~15% of all new cancer cases in 2015 (1,114,072 cases) [2]. Worldwide, the areas with a higher incidence of PC cases per 100,000 inhabitants are Oceania (101.9), North America (97.2), and Western Europe (94.9) [2–4]. In the Latin America region, PC was the most common malignancy diagnosed among males. The incidence of PC varied by 6-fold across this region during the period from 2003 to 2007. The highest age-standardized rates were observed in French Guyana (147.1) and Brazil (91.4) and the lowest ones were in Mexico (28.9) and Cuba (24.3). PC was one of the two leading causes of cancer deaths in males in Latin America, except in Chile, Argentina, Colombia, and El Salvador where it ranked third. Mortality rates varied by 4-fold, with the highest rates seen in Belize (28.9), Uruguay (21.8), and Cuba (24.1) and the lowest ones in Peru, Nicaragua, and El Salvador (rates between 6.8 and 9.7) [5]. In Ecuador 33% of all carcinoma diagnoses in males are prostate cancer, with an increase in the incidence from 23.7 per 100,000 inhabitants in 1985 to 54.4 in 2012 [2–4, 6]. Furthermore, the mortality rate associated with PC was 18.12 per 100,000 inhabitants in Ecuador in 2012 [2–4, 6, 7].

## 2. Genomic Landscape in Prostate Cancer

Due to the fact that cancer rates are increasing every year, new technologies are being applied to detect, manage, and treat it according to the patient. Consequently, in the last years, next-generation sequencing (NGS) has emerged in order to provide a comprehensive characterization in cancer and other diseases [8]. Moreover, NGS technology allows for the identification of base substitution, insertion-deletion, copy number variance, and structural alteration with good sensitivity in cancer [9]. Some studies using NGS have been carried out, obtaining relevant information in breast cancer, lung cancer, ovarian cancer, colorectal cancer, and melanoma [10]. It is important to mention that there are available various NGS techniques that could be applied in cancer, specifically in prostate cancer.

### 2.1. Whole-Genome Sequencing (WGS)

The WGS is the most complex technique based on the genomic DNA extraction from amplified and sequenced cancerous tissue giving, as a result, the somatic variations, such as mutations, insertions, and deletions, copy number variants, and rearrangements, among others. Nevertheless, the large amount of information that can be obtained with WGS, depending on the application (mutations, structural rearrangements, or others), needs time and a big coverage (100–200-fold coverage) that are translated into cost [11].

### 2.2. Whole-Exome Sequencing (WES)

WES is based on the protein coding genes (exome) present in 1-2% of the human genome. It can give huge amounts of variation data of all coding regions of known genes. WES is cheaper than WGS because it analyzes a sequence of interest 70–100-fold coverage in order to identify the presence of mutations [12].

### 2.3. RNA Sequencing

RNA sequencing needs an additional step, which is the reverse transcription from cDNA, but it can give important data in cancer analysis, such as mutations, dysregulated genes, variants, and the level of expression. However, RNA sequencing experiments can be affected by technical effects in the sequencing steps and by contamination [13].

### 2.4. Single-Cell RNA Sequencing

The heterogeneity of cancer can be completely understood by single-cell RNA sequencing and it can be used as an instrument for clinical decisions. Besides, it identifies driver mutations, differentially expressed genes when it is compared with normal tissue, and drug resistance and it can also suggest other therapeutic alternatives [11]. The technique includes the separation of tumor cells by laser capture, cell sorting (FACS), or microfluidics, followed by cell isolation.

Furthermore, PC has been considered a challenge in diagnosis and prognosis because of the highly heterogeneous nature and the limited sample of tumor tissue. Consequently, merging traditionally diagnostic methods with NGS can diagnose PC with greater accuracy. There are plenty of publications regarding studies in PC that reported using NGS through whole-genome and whole-exome technology and RNA sequencing, among others. For instance, Robinson et al. (2015) reported that specific AR mutations can be linked to clinical phenotypes in order to determine the mutations that are responsible for resistance to therapy and the 40%–60% of the cases showed abnormalities of AR, ETS genes, TP53, and PTEN [14]. Another study is the one published by Lohr et al. (2014) who characterized circulating tumor cells by WES, and the variants were in concordance with the tumor biopsies; the used methodology gave an alternative because of the type of sample even though both techniques must be combined in order to reduce false-positives and reveal new mutations [15]. Another study is the one published by Berger et al. (2011), where they identified 3,866 putative somatic base mutations, mutated genes, and MAGI2 genomic rearrangements that are directed to improve the diagnostic and patient stratification by PI3 pathways [16]. There are other PC studies that include different NGS techniques in order to determine the following: signaling pathways, genomic alterations, repair defects, and gene merging (Table 1).

The main genetic alterations reported for prostate cancer are as follows: (a) merging: the main merging described is the one between TMPRSS2 and ETS family verified by various NGS techniques achieving the same result, constituting a powerful tool for diagnostic PC [16–18]; (b) mutations: P53 mutation is linked in most tumor types including hereditary. It is involved in the response of cellular stress, cell survival, apoptosis, DNA repair, or change in the metabolism [11]; (c) pathways deregulations: there are studies that reported proliferative pathways that may promote cancer proliferation in the prostate. The AR abnormality is the most mentioned one in bibliography because it can modulate NCOA2, NCOR2, and regulatory elements like FOXA1 [16, 19–21]. Another signaling that can be found in prostate cancer is implied in cell growth and proliferation and apoptosis-like PI3K and PTEN [9]; and (d) variants and rearrangement: one variant described was at the 8q24 locus being a prostate cancer risk variant [22]. Furthermore, CHD1 regulates the chromatin state and its rearrangements are associated with more copy number variants [23].

## 3. The Steroid 5*α*-Reductase Type II Gene (SRD5A2) and Prostate Cancer Risk

The association of polymorphisms in the steroid 5*α*-reductase type II (SRD5A2) gene with prostate cancer risk in the high altitude mestizo Ecuadorian population was studied in 2009 by Paz-y-Miño et al. [24]. The steroid 5*α*-reductase type II enzyme is responsible for metabolizing the main androgenic hormone called testosterone that helps in the growth and development of the prostate. Due to the activity of the type II enzyme, testosterone is irreversibly converted into dihydrotestosterone (DHT), which is the reduced and metabolically more active form [25]. DHT then binds to the androgen receptor for the transactivation of androgen-sensitive elements as well as those that control cell proliferation. The SRD5A2 gene encodes the steroid 5*α*-reductase type II enzyme whose enzymatic activity may vary in such a way that it influences the incidence of PC [26].

The SRD5A2 gene plays an essential role in the induction of androgenic stimulation in the prostate and is highly polymorphic [27]. Hsing et al. suggest that genetic variations of this gene such as A49T and V89L are commonly studied and related to this type of cancer [26]. The V89L polymorphism, a missense mutation that substitutes leucine for valine at codon 89, identified by Makridakis et al., was reported to reduce in vivo steroid 5*α*-reductase activity [28]. Also, they found this substitution to be more common among Asians and believed this may explain the low risk for prostate cancer within this population. Another common polymorphism that has been reported to vary noticeably across populations is A49T, which results in an alanine residue at codon 49 being replaced with threonine. This missense substitution may enhance the conversion of testosterone into dihydrotestosterone because of increased enzymatic activity in vitro and has been associated with a higher risk of advanced prostate cancer in African American and Hispanic men living in the United States [29]. A total of 258 individuals, including 114 individuals with a previous diagnosis of prostate cancer and 144 control men, were analyzed to determine the association of SRD5A2 gene polymorphisms with the pathological characteristics of the tumor and the risk of PC in the high altitude mestizo Ecuadorian population.

The affected group had a median age of 70 years. 58% of the individuals were between 46 and 93 years old and 16% were older than 80 years. The pathological features of prostate tumors were quite variable, and the pathological stage of the tumor in most patients was moderate while 27% of the cases were diagnosed with advanced cancer. The 65% of the cases had serum prostate specific antigen levels greater than 10 ng/mL. The 23% of the individuals presented seminal vesicle invasion and 14% of the cases had positive surgical margins. The sixth level of Gleason grade is characterized because the cells are differentiated, in such a way that 59% of the cases have this score. There were significant differences between the tumor status and the presence of V89L polymorphism with the VV or VL genotype, where the LL genotype presented a highly significant reduction regarding the development of a high tumor stage (OR = 0.11, 95% CI = 0.04–0.27, and *P* < 0.001). While the higher pTNM stage (OR = 2.88, 95% CI = 1.15–7.21, and *P* = 0.039) and an elevated Gleason grade (OR = 3.15, 95% CI = 1.13–8.78, and *P* = 0.043) are associated with the A49T polymorphism.

Nam et al. and Li et al. showed that the V allele of the V89L polymorphism in the SRD5A2 gene was associated with PC risk because this V allele may encode for 5*α*-reductase variants with different activities, which are likely attributed to altered mRNA stability, which could alter the steroid 5*α*-reductase protein, leading to an increased cell division and, therefore, a higher likelihood of carcinogenesis [30, 31].

The genotype and allelic distribution of the V89L polymorphism in the Ecuadorian population support the hypothesis of the L allele having a protective status because of its suggested lower 5*α*-reductase enzymatic activity among men with the LL genotype [26, 28, 32, 33]. Other studies, however, have conversely found the LL genotype of the SRD5A2 V89L polymorphism to significantly increase the risk of PC [34–37]. Differences in genetic, dietary, and environmental factors among populations may explain the inconsistent results obtained in different studies. Our study also shows that men carrying the A49T variant have prostate tumors with a higher pathologic tumor-lymph node-metastasis (pTNM) stage (OR = 2.87; 95% CI = 1.15–7.21; *P* = 0.039) and a high Gleason grade (OR = 3.15; 95% CI = 1.13–8.78; *P* = 0.044). Our results agree with those of Jaffe et al., who reported the T allele to be linked to a greater frequency of extracapsular disease and a higher pTNM stage [38]. The genotype and allelic distribution are shown in Table 2, the OR test associated with polymorphisms is shown in Table 3, and the OR test associated with clinical data is shown in Table 4.

According to the 1000 Genomes' Project (Phase 3), the allele frequencies of the SRD5A2 V89L polymorphism are *G* = 0.255 and *C* = 0.745 in Colombians, *G* = 0.406 and *C* = 0.594 in Peruvians, *G* = 0.233 and *C* = 0.766 in Iberians, *G* = 0.389 and *C* = 0.611 in Japanese, *G* = 0.387 and *C* = 0.613 in Indians, *G* = 0.524 and *C* = 0.476 in Han Chinese, and *G* = 0.320 and *C* = 0.680 in the mestizo Ecuadorian population [39]. Also, the allele frequencies of the SRD5A2 A49T polymorphism are *C* = 0.979 and *T* = 0.021 in Colombians, *C* = 0.982 and *T* = 0.018 in Peruvians, *C* = 0.981 and *T* = 0.019 in Iberians, *C* = 1.000 and *T* = 0.000 in Japanese, *C* = 1.000 and *T* = 0.000 in Indians, *C* = 1.000 and *T* = 0.000 in Han Chinese, and *C* = 0.550 and *T* = 0.450 in the mestizo Ecuadorian population [39].

In conclusion, we found highly significant associations between two polymorphisms in the SRD5A2 gene. The V allele of the V89L polymorphism is associated with an increased risk, and the LL genotype has a protective role in the progression to a higher pTNM stage, while the TT homozygous genotype of the A49T polymorphism is associated with a higher pTNM stage and an elevated Gleason in the high altitude mestizo Ecuadorian population.

## 4. The Folate-Metabolizing MTHFR Gene and Prostate Cancer Risk

In 2013, López-Cortés et al. associated the folate-metabolizing genes with pathological characteristics of prostate cancer in the high altitude Ecuadorian mestizo population [40]. Regarding the folate cycle, the methylenetetrahydrofolate reductase (MTHFR), methionine synthase (MTR), and MTR reductase (MTRR) enzymes play an essential role in the folate metabolism [41], which is an important source for RNA and DNA synthesis and methylation [42]. The MTHFR enzyme controls a reaction of 5,10 methylenetetrahydrofolate to 5-methylenetetrahydrofolate [43–45], which is used as a methyl group donor for the remethylation of homocysteine to methionine, being a precursor of S-adenosylmethionine (SAM), essential in methylation of phospholipids, proteins, RNA, and DNA [41, 46, 47]. The remethylation of homocysteine to methionine is catalyzed by MTR in a reaction depending on vitamin B_12_ as an intermediary carrier of methyl group [48]. The MTR enzyme becomes inactive when the remethylation cofactor (vitamin B_12_) is oxidized by the MTRR enzyme [46, 49]. The MTRR enzyme catalyzes the regeneration of methylcobalamin, MTR cofactor, keeping the MTR active [50]. On the other hand, 5,10-methylenetetrahydrofolate is used by the thymidylate synthase enzyme in the methylation of deoxyuridylate to* deoxythymidylate*, which is a source of thymidine required for DNA synthesis and repair [46].

Folate deficiency is associated with the rise in DNA rupture, chromosome damage, and formation of micronucleus in lymphocytes. These effects may depend on hereditary defects or be acquired in the absorption and metabolism of folic acid [51]. The MTHFR C677T single nucleotide polymorphism has been identified in the alteration of the levels of folates and homocysteine [52], triggering an alteration of the functioning of expressed proteins [46]. The presence of the allelic variants of the MTHFR C677T polymorphism causes reduced enzymatic activity, alteration in the homocysteine levels, and folate concentration in plasma [46, 50]. The MTHFR C677T variant has been associated with neural tube defects, cerebrovascular diseases [53, 54], coronary artery disease, venous thrombotic disease, and the rise in the risk of developing ovary cancer [55], esophagus cancer [56], gastric cancer [57], and prostate cancer [58]. On the contrary, a low risk of developing leukemia and colorectal cancer has been observed [59, 60]. A retrospective case-control study was conducted to establish the frequency of the C677T polymorphism in the MTHFR gene related to the folate metabolism, DNA synthesis and methylation, and their association with pathological characteristics in the high altitude mestizo Ecuadorian individuals with PC. Two hundred fourteen individuals altogether were analyzed. One hundred samples were taken from individuals diagnosed with PC after going through prostatectomy between 2004 and 2006. One the other hand, the control group was made up of 110 healthy men.

About the clinical-pathological parameters, 61% of the individuals affected by PC were aged between 63 and 79 years, whereas 16% were older than 79 years. Regarding pathological tumor stage, 30% of the cases were diagnosed with advanced cancer. Forty-one percent of the individuals had a Gleason score between 7 and 10 (poorly differentiated carcinoma). Concerning the PSA levels, 65% of the cases had a level higher than 10 ng/mL. Fourteen percent of the individuals had positive surgical margins, whereas 23% of the cases showed positive invasion of the seminal vesicle. The Gleason grading system for PC is the dominant method around the world in research and daily practice. A common practice has been to translate Gleason score of 2–4 carcinoma as well-differentiated, Gleason score of 5-6 as moderately differentiated, and Gleason scores of 7–10 as poorly differentiated [61]. Because there was no significant risk between the CT + TT genotypes and the Gleason score of 2–4 versus 5-6 (OR = 0.9; *P* = 1) and because there was significant risk between the Gleason score of 2–4 versus 7–10 (OR = 5.2, *P* = 0.007), an association between the Gleason grade and the MTHFR C677T polymorphism was shown.

The MTHFR gene has been widely studied, and it was observed that the T/T677 genotype reduces the risk of colorectal cancer, acute lymphocytic leukemia, and malignant lymphoma [59, 62, 63]. Hypermethylation plays an important role in ontogenesis, silencing expressions of CpG islands in regions that promote tumor suppressor genes [64]. On the contrary, Heijmans et al. [47], Sharp and Little [65], Robien and Ulrich [66], Shen et al. [67], Song et al. [56], and Miao et al. [57] have observed association between the MTHFR C677T variant and different types of cancer [55]. For this reason, there is a continuous debate on the effects of the MTHFR C677T polymorphism on PC [41]. In our study, we found that the MTHFR C/T genotype is significantly risky in affected individuals with an OR of 2.2 (*P* = 0.008), being a possible association between this polymorphism and PC; therefore, these results were similar to those provided by Marchal et al. [68] and Van Guelpen et al. [69]. The genotype and allelic distribution are shown in Table 2, the OR test associated with polymorphisms is shown in Table 3, and the OR test associated with clinical data is shown in Table 4.

According to the 1000 Genomes' Project (Phase 3), the allele frequencies of the MTHFR C677T polymorphism are *G* = 0.457 and *A* = 0.543 in Colombians, *G* = 0.565 and *A* = 0.435 in Peruvians, *G* = 0.556 and *A* = 0.444 in Iberians, *G* = 0.620 and *A* = 0.379 in Japanese, *G* = 0.897 and *C* = 0.103 in Indians, *G* = 0.534 and *A* = 0.466 in Han Chinese, and *G* = 0.640 and *A* = 0.360 in the mestizo Ecuadorian population [39].

In conclusion, the association between Gleason grade, MTHFR gene, and prostate cancer is an important contribution to understanding the different genetic behavior of cancer between the high altitude mestizo Ecuadorian population and populations worldwide.

## 5. The Androgen Receptor (AR) Gene and Prostate Cancer Risk

In 2016, Paz-Y-Miño et al. positively associated the androgen receptor CAG repeat length polymorphism with the risk of prostate cancer in the high altitude Ecuadorian mestizo and indigenous populations [70]. This study was performed to determine the association between CAG and GGC repeats and the risk of PC and histopathological characteristics of prostate tumors. The prostate cell cycle is mediated by the interactions of the androgen receptor (AR) gene, which is located on chromosome Xq12 and encodes a protein that has three major functional domains: the N-terminal domain (NTD), DNA-binding domain, and ligand-binding domain. The NTD, encoded by exon 1, regulates the transactivation of target genes and contains two polymorphic trinucleotide repeats: CAG and GGC, encoding polyglutamine and polyglycine, respectively [71]. The length of the CAG repeats correlates inversely with the AR transactivation function [72, 73]. Moreover, Hakimi et al. and Irvine et al. associated a low number of CAG repeats with an increased risk of PC [74–78]. Furthermore, the variants reported in the CAG and GGC repeats are highly polymorphic and associated with ethnic factors; thus, it may be important to determine their association with PC in different populations. Trinucleotide repeats are associated with human diseases and microsatellite instability [79]. The last one affects gene expression and protein function [80]. In addition to PC, the polymorphic CAG repeats have been associated with skin disorders [81, 82], breast cancer, polycystic ovary disease, Kennedy syndrome [83, 84], azoospermia, and oligospermia [85]. Furthermore, the effects of the repetition sequence GGC polyglycine have been associated with hypospadias and cryptorchidism [86, 87]; however, its role in transcription is unclear.

According to Kittles et al. the CAG and GGC repeats specifically vary depending on the ethnic group [88, 89]. The normal distribution of the CAG repeats is reported in a range of 6–39, with an average of 19-20 in African Americans, 21-22 in Caucasians, 22-23 in Asians, and 23 in Hispanics [90]. However and regarding South American countries, Brazil reported an average of 20.65 CAGs [91]. Moreover, Madjunkova et al. reported a mean repeat length of 21.5 CAGs in patients with PC from Macedonia [92]. Beilin et al. examined prostate adenocarcinomas, and the number of CAG repeats ranged from 12 to 30 and averaged 20, which was similar to that in a healthy Brazilian population [91]. Paz-y-Miño et al. reported that the repeat range in the mestizo control group was 16–30, with an average of 22, resembling Asians and Caucasians. The study was performed to determine the association between CAG and GGC repeats and the risk of PC and histopathological characteristics of prostate tumors. A total of 334 individuals were analyzed; 108 mestizo patients had a clinical diagnosis of prostate adenocarcinoma and 148 mestizo patients were healthy. Additionally, 78 healthy indigenous individuals were analyzed to determine the variety of trinucleotide repeats between different ethnic groups. The patient group presented with 12–30 repetitions with an average of 20. The most common numbers of CAG repeats were 21 and 22 (15.7 and 12%, resp.). The mestizo control group had 16–30 repetitions with an average of 22. The most common numbers of CAG repeats were 22 (25.7%) and 25 (12.8%). In the indigenous population, the repeat size ranged from 18 to 29 CAGs, determining the highest average of the three groups corresponding to 24 repetitions. Thus, the most common numbers of CAG repeats were 24 and 26, both with 24.4%. Statistically significant differences (*P* < 0.001) in the distribution of these trinucleotides were demonstrated (≥22 CAGs versus ≤21 CAGs). In relation to tumor clinical characteristics, the presence of =21 CAGs showed significant association with tumor stage (OR, 4.75; 95% CI, 1.77–12.72; *P* < 0.05) and Gleason score (OR, 2.9; 95% CI, 1.1–7.66; *P* = 0.03) as in the ratio of risk of prostate cancer [70].

The ranges and averages of the CAG and GGC repeats in control mestizo population are similar to those of Asian and Caucasian-European populations. This may occur because the mestizo Ecuadorian population is considered a trihybrid, containing genes originated from America and descendants of Native Asians, Europeans, and Africans [93]. Other polymorphisms associated with PC in Ecuador also showed similar frequencies to Asians [24, 40]. Additionally, these results were consistent with the average in cases (19 CAGs) and controls (19-20 CAGs) in African Americans (17 CAGs) and in both cases and controls (21.95 CAGs) in Australians [94].

Finally, these results indicated that in mestizos the PC risk increased 2.99 times in males with ≤21 CAGs. By contrast, some studies did not identify an association with this repetition [43, 95, 96]. Several studies reported no association between the GGC repeat lengths, the PC risk, and pathological characteristics [34], stating that there were no significant differences between cases and controls [97]. Similarly, the risk of PC and the tumor characteristics did not differ in relation to the number of GGC repeats in this study [93].

## 6. Conclusions

Cancer research has evolved in parallel with cutting-edge technologies, leading to the development of a personalized genomic-based therapy. This tailored treatment not only takes into account the clinical aspects of each patient but also, and most importantly, the molecular characteristics of their tumors. Thus, to offer a precise anticancer therapy, personalized oncology identifies druggable cancer driver proteins based on their genomic alterations and differences between human populations. For instance, the 1000 Genomes' Project (Phase 3) demonstrates that the allele frequencies of the SRD5A2 V89L, SRD5A2 A49T, and MTHFR C677T genetic variants differ among the Latin American (Ecuador, Colombia, and Peru), Caucasian (Spain), and Asian (Japan, India, and China) populations. In conclusion, in order to implement successful pharmacogenomics tests at the hospitals in Ecuador, it is important to understand the genetic variability of the mestizo population. Likewise, genetic polymorphisms in the MTHFR, SRD5A2, and AR genes are associated with PC risk in high altitude mestizo Ecuadorian population.

## Figures and Tables

**Table 1:** Different NGS techniques used in prostate cancer studies.

Finding	NGS technology	Year	Reference
Deletion at chromosome 3p14 implicates FOXP1, RYBP, and SHQ1 as potential cooperative tumor suppressors	WES	2010	[21]
TMPRSS2-ERG, CTAGE5-KHDRBS3, and USP9Y-TTTY15 fusions, long noncoding RNAs (long ncRNAs), alternative splicing, and somatic mutations	RNA sequencing WGS	2011, 2012	[16, 18]
SPOP, FOXA1, and MED12 mutations	WES	2012	[20]
Variant at 8q24 and HOXB13	WGS	2012	[22]
Rearrangements, translocations, and deletions	WGS	2013	[23]
ADP-regulated signaling pathways-inhibitions of Wnt/B catenin signaling pathways	RNA sequencing	2014	[17]
Heterogeneity of AR gene expression, mutations, and splicing variants	Single-cell RNA seq	2015	[19]

**Table 2:** Genotype distribution and allele frequency of polymorphisms in SRD5A2, MTHFR, and AR genes in high altitude Ecuadorian population with prostate cancer.

Gene	Polymorphism	Genotype	Genotypic frequency			Allele frequency		
			Control	Case	All	Control	Case	All
SRD5A2	A49T	A/A	0.347	0.289	0.322	0.674	0.553	0.627
		A/T	0.653	0.526	0.597			
		T/T	0.000	0.184	0.081	0.326	0.447	0.373
	V89L	V/V	0.153	0.404	0.264	0.469	0.684	0.564
		V/L	0.632	0.561	0.601			
		L/L	0.215	0.035	0.136	0.531	0.316	0.436

MTHFR	C677T	C/C	0.473	0.288	0.383	0.732	0.639	0.687
		C/T	0.518	0.702	0.607			
		T/T	0.009	0.010	0.009	0.268	0.361	0.313

AR	CAG repeats	≥22 CAGs	0.639	0.372	0.484			
		≤21 CAGs	0.361	0.628	0.516			
	GGC repeats	≥17 GGCs	0.389	0.281	0.329			
		≤16 CAGs	0.611	0.719	0.671			

**Table 3:** Association between genetic polymorphisms and prostate cancer risk among cases and controls.

Gene	Polymorphism	Genotype	Case versus control		
			OR	95% CI	*P* value
SRD5A2	A49T	A/A			Reference
		A/T	0.97	0.56–1.67	1
		T/T	0.40	0.31–0.52	0.000
		A/T + T/T	1.31	0.77–2.22	0.394
	V89L	V/V			Reference
		V/L	0.34	0.19–0.61	
		L/L	0.06	0.02–0.20	
		V/L + L/L	0.27	0.15–0.48	0.000

MTHFR	C677T	C/C			Reference
		C/T	2.22	1.26–3.92	0.008
		T/T	1.73	0.11–28.73	1
		C/T + T/T	2.21	1.26–3.89	0.008

AR	CAG repeats	≥22 CAGs			Reference
		≤21 CAGs	2.99	1.79–5.01	0.000

OR, odds ratio.

**Table 4:** Association of prostate cancer risk with genotype polymorphisms and clinical features.

Gene	Polymorphism	Genotype	Gleason score		OR	95% CI	*P* value	Tumor stage		OR	95% CI	*P* value
			2–6	7–10				T1-T2b	T2c-T4			
SRD5A2	A49T	A/A	20	6	Reference			17	10	Reference		
		A/T + T/T	36	34	3.15	1.13–8.78	0.044	26	44	2.88	1.15–7.21	0.039
	V89L	V/V	19	24	Reference			8	36	Reference		
		V/L + L/L	37	16	0.34	0.15–0.79	0.020	36	17	0.11	0.04–0.27	0.000

MTHFR	C677T	C/C	25	5	Reference			12	15	Reference		
		C/T + T/T	32	34	5.31	1.81–15.56	0.003	33	35	0.85	0.35–2.08	0.895

AR	CAG repeats	≥22 CAGs	19	8	Reference			19	8	Reference		
		≤21 CAGs	27	33	2.90	1.10–7.66	0.05	20	40	4.75	1.77–12.72	0.003

OR, odds ratio.
